# Chemical profile dataset of *Cornus officinalis* from multiple sources using HPLC/MS

**DOI:** 10.1016/j.dib.2019.104401

**Published:** 2019-08-13

**Authors:** Arielle E. Sharp-Tawfik, Brant R. Burkhardt

**Affiliations:** University of South Florida, Department of Cell Biology, Microbiology and Molecular Biology, USA

**Keywords:** *Cornus officinalis*, HPLC, Mass spectrometry, Chemical analysis, Traditional Chinese medicine

## Abstract

This article displays raw data linked to the research article “Compositional Analysis and Biological Characterization of Cornus officinalis on Human 1.1B4 Pancreatic β Cells” [1]. This data was generated by utilizing HPLC/(+and -)ESI-MSn on *Cornus officinalis* (CO) from four independent sources [1]. The aim was to identify the chemical profile of CO from multiple sources to compare the similarities and differences resulting from various processing methods, and compile a list of known and novel constituents to elucidate the bioactive ingredients. This report contains the full chromatogram and a raw list of the constituents found in CO including chemical name, retention time, and molecular weight from all four sources. All data from HPLC/MS analysis is raw and unprocessed.

**Table:** Specifications Table

Subject area	*Chemistry*
More specific subject area	*Spectroscopy*
Type of data	Tables, Figures
How data was acquired	HPLC/(+ and −)ESI-MSn
Data format	*Raw*
Experimental factors	The chemical profile of *Cornus officinalis* from four independent sources (sources 1–4) were individually analyzed by HPLC/MS.
Experimental features	Approximately 0.5 g of dried powder from each source were extracted with 1.5 mL H_2_O + 1.5 mL methanol. After sonication and centrifugation, some of the supernatant was transferred to autosampler vials and analyzed via C8 HPLC/(+ and −)ESI-MSn.
Data source location	Cell Biology, Microbiology and Molecular Biology. University of South Florida, Tampa, Florida, United States.
Data accessibility	Data is within this article
Related research article	Sharp-Tawfik, A.E., Coiner, A.M., MarElia, C.B., Kazantzis, M., Zhang, C., and Burkhardt, B.R. (2019). Compositional analysis and biological characterization of Cornus officinalis on human 1.1B4 pancreatic beta cells. Mol Cell Endocrinol *494*, 110491 [1].

**Table:** 

**Value of the data** • This is the first dataset to compare the chemical profile of CO from 4 sources to display similarities and differences in profile relating to various processing methods and independent sources • This dataset could provide insight for investigators to identify the optimum composition and bioactive compounds for treatment of specific diseases using *Cornus officinalis.* • This dataset is valuable in showing the importance of HPLC/MS to analyze the extract at use to verify active ingredients.

## 1 Data

**Fig. 1:**
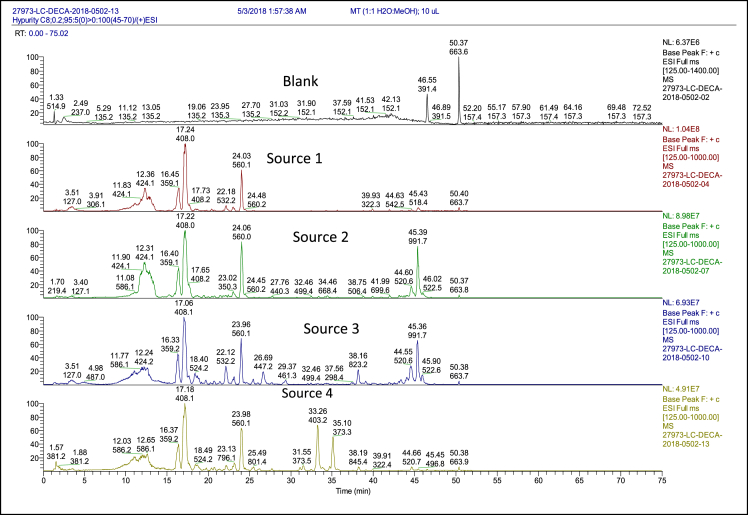
HPLC/MS Chromatogram from all four sources of Cornus officinalis. The blank (1:1 H_2_O:MeOH) is shown at the top of this figure.

HPLC/(+ and −)ESI-MSn was performed on four sources of CO obtained from independent companies of which all follow good manufacturing processes (GMP) and quality control. Sources 1–4 include Guangdong Yifang Pharmaceutical Co., Ltd, Treasure of the East Tianjiang Pharmaceutical Co., Ltd, Min Tong Co, and Evergreen Herbs International, LLC. The individual preparations of CO differed by extraction/purification method. Source 1 has been processed using rice wine followed by water extraction to increase active ingredients. Sources 2–4 were all processed using traditional water-extraction followed by high pressure, low temperature vacuum extraction and granulation. After vacuum extraction, sources 1 and 2 performed a spray drying process to protect the active ingredients followed by a re-introduction of volatile oils by source 1. During the granulation process, source 3 utilizes non-GMO cornstarch as an addition to prevent caking and ensure stability of the extract. All four sources perform quality control assessments to examine bacteria levels, heavy metals, and pesticide residue. The chromatogram showing peaks of all compounds over 75 minutes from all four companies is displayed in Fig. 1. The Supplementary Tables 1 and 2 include the raw ion-peak detected in all samples with identified chemical name (if known), molecular weight, retention time and peak area.

## 2 Experimental design, materials, and methods

### 2.1 HPLC/MS

Approximately 0.5 g of dried powder were extracted with 1.5 mL H2O + 1.5 mL methanol. After sonication and centrifugation, some of the supernatant was transferred to autosampler vials and analyzed via C8 HPLC/(+ and −)ESI-MSn. The analysis was carried out on Agilent (Palo Alto, CA) 1100 series system consisting of G1313A autosampler, G1322A degasser and G1312A binary pump followed by ThermoFinnigan (now ThermoFisher Scientific; San Jose, CA) LCQ DECA quadrupole ion trap mass spectrometer with electrospray ionization (ESI) operating with XCALIBUR 2.0.7. SP1. The chromatographic parameters were Thermoscientific Hypurity C8 (5 μm; 2.1 × 100 mm + guard column). Mobile phase (A): water + 0.2% acetic acid and mobile phase (B): methanol + 0.2% acetic acid with the following gradient: 0.2 mL/min: A:B(min) = 95:5(0) => 0:100(45–70) => 95:5(80–90). The + ESI parameters were as follows: spray voltage = 4.0 kV; heated capillary voltage = −18 V; tube lens offset = +8 V. The –ESI parameters were: spray voltage = 4.5 kV; heated capillary voltage = +6 V; tube lens offset = −2 V. The duration was 75 min with 4 scan events. The spectra of ion-peaks were examined and from those, related ions were plotted to create new ion-peaks in order to determine which ions correlated with each other. An ion-peak which was characteristic of a compound peaks was then integrated for the different samples.
