# Supplementary material for: Chemical profile dataset of Cornus officinalis from multiple sources using HPLC/MS
Source: Data Brief. 2019 Aug 13;25:104401. doi: 10.1016/j.dib.2019.104401 (PMC6722233; doi:10.1016/j.dib.2019.104401)
Supplement: Multimedia component 1 [file mmc1.docx]

|  | |  |  |  |  | **Peak Areas** |  |  |
| --- | --- | --- | --- | --- | --- | --- | --- | --- |
| **Compound** | | **MW** | **Avg RT** | **Blank** | **Source 1** | **Source 2** | **Source 3** | **Source 4** |
| MW 296 (296 - 134 = 162 u; glycoside) | | 296 | 1.62 | 0 | 13,832,156 | 6,700,066 | 33,113,794 | 27,409,212 |
| Tartaric Acid | | 150 | 1.91 | 0 | 31,224,882 | 33,226,948 | 16,431,801 | 21,116,300 |
| Malic Acid | | 134 | 1.98 | 0 | 281,637,713 | 274,318,527 | 205,938,480 | 217,195,643 |
| MW 412-A | | 412 | 2.08 | 0 | 4,066,596 | 1,051,293 | 12,309,194 | 10,074,287 |
| MW 192 | | 192 | 2.09 | 0 | 40,008,595 | 39,824,268 | 21,547,821 | 20,567,340 |
| MW 232 | | 232 | 2.20 | 0 | 1,762,474 | 1,102,535 | 215,891 | 495,056 |
| MW 250 | | 250 | 2.22 | 48,765 | 5,508,756 | 1,835,805 | 2,940,419 | 6,570,247 |
| Malic Acid, methyl Ester | | 148 | 2.52 | 0 | 1,256,944 | 1,409,859 | 2,489,192 | 3,278,644 |
| MW 460 | | 460 | 2.58 | 0 | 10,661,962 | 16,702,025 | 10,483,567 | 16,196,936 |
| MW 272 (or fragment ion of MW 362) | | 272/362 | 2.59 | 0 | 9,386,936 | 17,846,937 | 18,558,787 | 17,557,499 |
| MW 304 | | 304 | 2.60 | 0 | 19,641,201 | 21,867,479 | 16,825,341 | 10,567,863 |
| Mw 362 | | 362 | 2.66 | 0 | 55,372,838 | 110,763,396 | 72,332,398 | 65,612,961 |
| 5-Hydroxymethyl-2-furaldehyde | | 126 | 2.76 | 0 | 1,326,948 | 819,354 | 269,215 | 373,970 |
| Gallic Acid | | 170 | 2.85 | 0 | 91,967,871 | 71,916,684 | 42,669,645 | 42,929,502 |
| MW 644 | |  | 3.01 | 0 | 7,226,495 | 20,093,209 | 3,130,780 | 4,479,033 |
| MW 344 | | 222 | 3.06 | 0 | 10,242,918 | 7,664,852 | 8,841,733 | 9,156,399 |
| MW 524 | |  | 3.33 | 0 | 6,118,012 | 2,399,702 | 15,631,439 | 10,964,806 |
| MW 162 | | 162 | 3.59 | 0 | 16,193,796 | 2,262,074 | 6,309,106 | 1,136,205 |
| MW 348 | |  | 3.67 | 0 | 5,324,92 | 829,212 | 2,549,84 | 1,335,827 |
| MW 478 | |  | 3.84 | 0 | 18,180,962 | 9,641,438 | 54,436,516 | 34,334,000 |
| MW 312: only m/z 311 | | 312 | 3.93 | 0 | 3,497,493 | 26,028,950 | 6,047,348 | 13,746,636 |
| Mw 484 | | 484 | 4.04 | 0 | 13,157,565 | 16,053,872 | 8,034,096 | 5,913,704 |
| MW 412-B | | 412 | 4.13 | 0 | 3,523,259 | 9,850,859 | 4,588,337 | 4,512,690 |
| MW 480 | |  | 4.14 | 0 | 19,994,810 | 9,012,360 | 6,885,878 | 8,799,821 |
| MW 514 | |  | 4.18 | 0 | 28,755,771 | 75,110,897 | 36,002,476 | 46,012,265 |
| Mw 172 | | 172 | 4.26 | 0 | 2,918,569 | 5,189,432 | 1,844,074 | 1,597,509 |
| MW 154 | | 154 | 5.07 | 0 | 10,527,834 | 14,364,847 | 2,305,991 | 7,759,602 |
| MW 138 | | 138 | 7.94 | 0 | 2,101,733 | 1,225,432 | 1,136,111 | 1,391,745 |
| MW 242 | | 242 | 8.63 | 0 | 9,042,167 | 745,164 | 18,257,798 | 2,406,101 |
| MW 514 | |  | 8.88 | 0 | 2,592,421 | 4,047,256 | 6,375,391 | 4,724,002 |
| MW 510; m/z 329 fragment ion) | | 510 | 8.95 | 0 | 37,428,785 | 21,671,262 | 26,014,009 | 20,472,983 |
| MW 296 | | 296 | 9.45 | 0 | 200,430 | 3,359,537 | 1,188,555 | 2,569,155 |
| MW 404, m/z 463 [M+59]- | | 464/404 | 9.96 | 0 | 3,850,311 | 2,143,633 | 1,359,602 | 754,404 |
| MW 138 | | 138 | 10.09 | 0 | 4,078,322 | 3,731,751 | 1,984,297 | 2,083,339 |
| MW 444 | | 444 | 10.09 | 0 | 334,120 | 469,712 | 774,405 | 272,116 |
| MW 404 or m/z 403 fragment ion | | 464/404 | 10.59 | 0 | 5,115,291 | 523,876 | 15,784,262 | 3,301,860 |
| MW 406; m/z 405 | | 406 | 10.97 | 0 | 9,946,800 | 4,482,774 | 14,913,832 | 13,556,098 |
| m/z 285 | |  | 11.05 | 0 | 3,734,017 | 235,475 | 2,999,356 | 3,745,205 |
| MW 388, 448, m/z 447 | | 388/448 | 11.14 | 0 | 5,258,271 | 1,444,435 | 7,267,743 | 8,894,790 |
| MW 474, m/z 473 | | 474 | 11.17 | 0 | 1,365,895 | 1,903,028 | 8,993,900 | 6,705,671 |
| MW 624 | | 624 | 11.33 | 0 | 0 | 10,278,288 | 824,904 | 1,172,732 |
| MW 312 | | 312 | 11.36 | 0 | 8,059,454 | 95,763,298 | 61,040,772 | 55,240,248 |
| m/z 179 fragment of MW 312 | | 312 | 11.36 | 0 | 12,401,984 | 22,417,772 | 18,049,632 | 11,040,746 |
| m/z 179 fragment ion of MW 312 | | 180 | 11.39 | 0 | 8,852,168 | 24,066,121 | 13,703,776 | 11,958,374 |
| m/z 315 | |  | 11.52 | 0 | 3,141,513 | 1,232,510 | 4,730,323 | 9,279,958 |
| MW 568 (diglycoside form of morroniside likely) | | 568 | 11.55 | 0 | 276,829,638 | 71,699,096 | 200,489,094 | 144,177,727 |
| m/z 285 | |  | 11.75 | 0 | 1,014,716 | 53,122 | 1,040,060 | 1,726,974 |
| MW 388, 448, m/z 447 | | 388/448 | 11.76 | 0 | 1,783,840 | 340,098 | 3,607,018 | 3,379,018 |
| MW 450, m/z 449 | | 450 | 11.77 | 0 | 9,773,269 | 6,771,517 | 4,319,509 | 1,621,399 |
| MW 478 | |  | 12.03 | 0 | 13,653,553 | 3,880,155 | 15,988,878 | 14,884,928 |
| MW 444 | | 444 | 12.20 | 0 | 6,763,998 | 8,740,535 | 5,198,417 | 3,284,953 |
| MW 522, m/z 521 | | 522 | 12.21 | 0 | 4,654,687 | 1,435,175 | 7,396,622 | 6,736,230 |
| Morroniside: m/z 405 and 465 have different profiles | | 406 | 12.30 | 0 | 335,572,983 | 357,757,725 | 117,305,564 | 65,387,594 |
| m/z 507 fragment ion of MW 568 | |  | 12.57 | 0 | 22,339,903 | 4,550,158 | 24,657,321 | 20,070,826 |
| m/z 285 | |  | 12.59 | 0 | 912,186 | 249,568 | 1,953,776 | 2,030,289 |
| MW 388, 448, m/z 447 | | 388/448 | 12.61 | 0 | 3,308,572 | 711,186 | 2,718,793 | 2,939,897 |
| Loganic Acid | | 376 | 12.88 | 0 | 164,487,735 | 175,555,555 | 65,735,429 | 59,860,735 |
| MW 522, m/z 521 | | 522 | 13.26 | 0 | 11,941,323 | 2,908,680 | 11,098,338 | 9,446,788 |
| MW 514, has m/z 271 fragment ion | |  | 13.32 | 0 | 101,545,190 | 190,271,620 | 184,671,340 | 207,628,538 |
| MW 388, 448, m/z 447 | | 388/448 | 13.33 | 0 | 1,115,205 | 412,386 | 2,146,921 | 1,568,774 |
| m/z 285 | |  | 13.36 | 0 | 334,388 | 48,785 | 1,474,173 | 1,359,687 |
| Mw 484 | | 484 | 13.51 | 0 | 10,708,790 | 18,989,760 | 16,760,507 | 9,484,066 |
| MW 636, m/z 635.1 | | 636 | 13.71 | 0 | 2,098,943 | 5,454,250 | 4,435,560 | 1,480,303 |
| Mw 636 | | 636 | 13.71 | 0 | 2,036,392 | 5,238,206 | 4,541,134 | 1,508,684 |
| MW 314 | | 314 | 13.72 | 0 | 349,225 | 2,085,853 | 7,964,629 | 731,700 |
| MW 180 | | 180 | 13.78 | 0 | 14,954,545 | 47,565,619 | 15,780,253 | 17,872,053 |
| MW 180 (m/z 179, 215/217) | | 180 | 13.80 | 0 | 13,044,260 | 44,068,423 | 15,699,506 | 17,073,713 |
| Mw 492, m/z 491 | | 492 | 14.01 | 0 | 0 | 0 | 1,390,840 | 546,541 |
| m/z 315 | |  | 14.28 | 0 | 965,798 | 36,412 | 1,324,531 | 2,444,939 |
| MW 580 | | 580 | 14.32 | 0 | 26,339,850 | 8,400,373 | 36,071,059 | 29,392,059 |
| MW 684 | | 684 | 14.33 | 0 | 56,196,833 | 6,983,250 | 138,471,820 | 55,958,527 |
| MW 506 | | 506 | 14.35 |  | 1,656,213 | 570,953 | 4,025,350 | 763,346 |
| MW 540 | | 540 | 14.40 | 0 | 7,358,057 | 2,718,298 | 1,766,911 | 781,498 |
| Mw 492, m/z 491 | | 492 | 14.59 | 0 | 1,336,467 | 981,296 | 2,788,131 | 1,552,358 |
| m/z 163 fragment ion of MW 296 | | 296 | 14.69 | 0 | 615,477 | 6,652,723 | 3,480,298 | 4,508,909 |
| MW 626, m/z 625 | | 626 | 14.71 | 0 | 481,739 | 2,211,384 | 546,986 | 695,123 |
| MW 522, m/z 521 | | 522 | 14.75 | 0 | 38,669,184 | 37,296,643 | 54,182,829 | 21,892,694 |
| Mw 296 (has a m/z 591 [2M-H]- | | 296 | 14.83 | 0 | 6,817,296 | 58,219,246 | 39,771,967 | 39,912,792 |
| MW 198 or m/z 197 fragemt ion | | 198 | 14.91 | 0 | 706,978 | 367,019 | 260,137 | 340,944 |
| Mw 492, m/z 491 | | 492 | 15.05 | 0 | 7,205,134 | 4,114,810 | 15,828,117 | 7,341,934 |
| MW 636, m/z 635.1 | | 636 | 15.09 | 0 | 2,295,872 | 6,740,473 | 2,226,054 | 2,410,665 |
| Mw 636 | | 636 | 15.09 | 0 | 2,383,586 | 7,108,586 | 2,292,687 | 2,308,709 |
| MW 478 | |  | 15.12 | 0 | 3,257,747 | 1,261,471 | 1,866,456 | 1,738,619 |
| 7-O-Methylloganic acid | | 390 | 15.32 | 0 | 18,554,399 | 31,654,305 | 15,347,670 | 11,655,216 |
| MW 390 | | 390 | 15.32 | 0 | 18,424,901 | 31,830,682 | 15,347,670 | 11,916,487 |
| Tellimagrandin I | | 786 | 15.43 | 0 | 1,567,527 | 3,705,779 | 973,939 | 907,020 |
| Mw 492, m/z 491 | | 492 | 15.46 | 0 | 10,025,164 | 9,381,595 | 17,066,428 | 12,110,494 |
| MW 308 (has m/z 343/345 ion) | | 308 | 15.55 | 0 | 5,747,909 | 2,588,481 | 10,581,357 | 3,255,752 |
| MW 450, m/z 449 | | 450 | 15.68 | 0 | 3,842,974 | 8,035,365 | 3,378,750 | 5,383,125 |
| MW 390 | | 390 | 15.76 | 0 | 483,625 | 1,208,285 | 1,268,030 | 1,215,893 |
| MW 390, m/z 389 | |  | 15.81 | 0 | 343,629 | 1,391,899 | 1,277,838 | 1,333,222 |
| MW 388, 448, m/z 447 | | 388 | 15.96 | 0 | 22,135,377 | 13,253,888 | 6,542,718 | 5,778,453 |
| MW 226 | | 226 | 16.00 | 0 | 2,629,674 | 4,314,343 | 3,039,928 | 2,322,145 |
| MW 326 | | 326 | 16.01 | 0 | 666,988 | 3,127,573 | 3,179,637 | 1,395,420 |
| MW 506 | | 506 | 16.06 |  | 1,873,075 | 1,773,394 | 5,197,348 | 2,121,419 |
| MW 522, m/z 521 | | 522 | 16.25 | 0 | 30,348,051 | 19,274,881 | 36,925,579 | 18,337,413 |
| m/z 315 | |  | 16.32 | 0 | 0 | 268,352 | 557,259 | 589,144 |
| Sweroside | | 358 | 16.40 | 0 | 50,296,630 | 39,349,174 | 23,917,579 | 17,361,716 |
| MW 450, m/z 449 | | 450 | 16.44 | 0 | 2,439,095 | 6,075,381 | 2,040,682 | 2,991,052 |
| Mw 492, m/z 491 | | 492 | 16.49 | 0 | 2,797,472 | 2,263,957 | 5,002,499 | 2,453,918 |
| MW 198 or m/z 197 fragemt ion | | 198 | 16.53 | 0 | 704,965 | 2,135,762 | 1,812,559 | 2,059,501 |
| MW 636, m/z 635.1 | | 636 | 16.53 | 0 | 3,361,650 | 7,311,538 | 5,801,178 | 2,772,513 |
| Mw 636 | | 636 | 16.53 | 0 | 3,483,537 | 7,308,405 | 5,674,680 | 2,631,573 |
| MW 326 | | 326 | 16.58 | 0 | 421,978 | 4,796,426 | 2,683,880 | 3,267,753 |
| MW 314 | | 314 | 16.62 | 0 | 1,240,423 | 8,496,681 | 1,058,399 | 8,291,694 |
| MW 464 | | 464 | 16.65 | 0 | 1,223,686 | 396,651 | 343,888 | 209,607 |
| MW 666, m/z 665.2 | | 666 | 16.68 | 0 | 2,933,602 | 10,742,279 | 10,554,901 | 8,475,516 |
| MW 666 | | 666 | 16.68 | 0 | 2,883,898 | 11,421,052 | 10,999,846 | 9,233,463 |
| Mw 492, m/z 491 | | 492 | 16.81 | 0 | 2,237,849 | 1,762,121 | 5,502,847 | 3,077,238 |
| MW 482 or MW 704 | |  | 16.83 | 0 | 1,021,445 | 1,919,152 | 2,217,095 | 1,087,819 |
| MW 552 | | 552 | 16.87 | 0 | 3,229,649 | 1,013,806 | 1,527,304 | 1,001,458 |
| MW 358 isomer | | 358 | 16.91 | 0 | 2,386,905 | 1,491,086 | 865,848 | 1,143,860 |
| MW 426 | | 426 | 17.07 | 0 | 3,005,536 | 4,818,063 | 2,377,657 | 1,021,927 |
| MW 390 Loganin; see below | |  | 17.16 | 0 | 5,759,717 | 4,630,222 | 4,505,577 | 3,926,306 |
| Loganin, m/z 449 BP | | 390 | 17.21 | 0 | 198,057,241 | 142,754,143 | 90,378,512 | 67,218,462 |
| MW 560 (m/z 595 [M+Cl]-) | | 560 | 17.40 | 0 | 3,089,777 | 2,990,389 | 2,027,543 | 1,549,356 |
| Mw 492, m/z 491 | | 492 | 17.41 | 0 | 2,006,372 | 1,187,177 | 4,478,380 | 2,049,922 |
| MW 624 | | 624 | 17.50 | 0 | 1,132,534 | 581,624 | 1,289,964 | 931,341 |
| MW 506 | | 506 | 17.55 |  | 2,155,954 | 1,042,984 | 6,266,622 | 1,850,033 |
| MW 522, m/z 521 | | 522 | 17.57 | 0 | 5,585,861 | 3,082,430 | 10,799,009 | 4,162,031 |
| MW 482 or MW 704 | |  | 17.59 | 0 | 1,589,567 | 1,955,264 | 3,729,312 | 2,573,391 |
| MW 474, m/z 473 | | 474 | 17.63 | 0 | 921,027 | 300,120 | 2,751,373 | 621,458 |
| MW 552 | | 552 | 17.65 | 0 | 13,713,813 | 1,720,125 | 9,118,426 | 8,802,696 |
| MW 668, m/z 667; hump | | 668 | 17.66 | 0 | 6,086,133 | 1,950,214 | 22,818,238 | 6,980,550 |
| MW 666, m/z 665.2 | | 666 | 17.71 | 0 | 1,696,882 | 2,186,704 | 2,638,753 | 2,052,935 |
| MW 666 | | 666 | 17.71 | 0 | 1,702,825 | 2,135,234 | 2,705,302 | 2,420,902 |
| MW 164 | | 164 | 17.72 | 0 | 634,937 | 2,819,313 | 403,287 | 536,335 |
| MW 326 | | 326 | 17.74 | 0 | 263,970 | 1,198,537 | 521,285 | 743,055 |
| MW 540 | | 540 | 18.14 | 0 | 289,160 | 1,770,336 | 93,096 | 116,485 |
| MW 552 | | 552 | 18.19 | 0 | 2,669,795 | 956,756 | 2,810,189 | 1,442,213 |
| MW 164 (m/z 199/201 ion) | | 164 | 18.21 | 0 | 12,568,275 | 17,343,834 | 10,701,502 | 12,435,790 |
| MW 478 | |  | 18.25 | 0 | 1,120,823 | 226,086 | 241,071 | 361,658 |
| MW 390, m/z 389 | |  | 18.42 | 0 | 1,179,870 | 646,747 | 4,197,935 | 2,908,503 |
| MW 390 | | 390 | 18.42 | 0 | 1,277,699 | 523,921 | 4,198,926 | 2,565,845 |
| MW 474, m/z 473 | | 474 | 18.46 | 0 | 1,236,992 | 483,528 | 3,492,386 | 865,062 |
| MW 138 or m/z 137 fragment ion | | 138 | 18.49 | 0 | 116,662 | 2,663,650 | 2,194,392 | 349,428 |
| MW 506 | | 506 | 18.62 |  | 80,758,197 | 58,270,620 | 215,696,797 | 98,683,624 |
| MW 522, m/z 521 | | 522 | 18.67 | 0 | 1,076,708 | 326,502 | 513,196 | 160,606 |
| MW 308 (does not have m/z 343/345 ion) | | 308 | 18.83 | 0 | 1,368,660 | 3,270,589 | 2,367,778 | 2,557,918 |
| MW 390, m/z 389 | |  | 18.87 | 0 | 704,437 | 479,014 | 2,210,445 | 955,578 |
| MW 390 | | 390 | 18.87 | 0 | 347,520 | 349,244 | 1,657,071 | 658,519 |
| MW 164 | | 164 | 18.88 | 0 | 647,176 | 1,499,728 | 881,547 | 1,105,507 |
| MW 474, m/z 473 | | 474 | 18.90 | 0 | 2,029,482 | 715,964 | 3,695,173 | 1,350,530 |
| MW 560 (m/z 595 [M+Cl]-) | | 560 | 18.91 | 0 | 11,941,233 | 17,285,823 | 13,102,080 | 9,259,562 |
| MW 464 | | 464 | 18.94 | 0 | 2,272,101 | 10,023,102 | 4,515,295 | 4,567,836 |
| MW 540 | | 540 | 18.96 | 0 | 468,503 | 670,818 | 199,422 | 142,051 |
| MW 510 | | 510 | 19.23 | 0 | 305,044 | 869,316 | 289,500 | 206,330 |
| MW 480 | |  | 19.25 | 0 | 697,922 | 830,811 | 938,011 | 470,851 |
| MW 478 | |  | 19.25 | 0 | 2,975,600 | 3,968,087 | 4,607,537 | 2,609,456 |
| MW 522, m/z 521 | | 522 | 19.36 | 0 | 1,868,217 | 1,823,216 | 757,736 | 570,017 |
| MW 534 (2 isomers merged) | | 534 | 19.37 | 0 | 2,349,177 | 1,487,663 | 3,904,889 | 2,050,894 |
| MW 404 | | 404 | 19.41 | 0 | 9,423,411 | 16,007,368 | 6,922,039 | 5,986,470 |
| MW 372 | | 372 | 19.41 | 0 | 419,143 | 898,529 | 368,411 | 544,515 |
| MW 542 | | 542 | 19.49 | 0 | 1,537,804 | 2,817,037 | 1,110,066 | 1,238,824 |
| MW 474, m/z 473 | | 474? | 19.51 | 0 | 2,192,667 | 1,517,104 | 7,632,409 | 2,579,212 |
| MW 622 | | 622 | 19.69 | 0 | 2,384,695 | 952,408 | 23,308,503 | 3,925,327 |
| MW 478 | |  | 19.70 | 0 | 2,275,944 | 3,938,472 | 3,967,463 | 1,421,116 |
| MW 390 | | 390 | 19.70 | 0 | 6,797 | 7,228 | 873,647 | 271,083 |
| MW 390, m/z 389 | |  | 19.73 | 0 | 0 | 0 | 1,031,071 | 416,819 |
| MW 506 | | 506 | 19.76 |  | 25,075,916 | 16,242,834 | 69,188,370 | 31,406,877 |
| MW 522, m/z 521 | | 522 | 19.77 | 0 | 2,608,765 | 2,004,470 | 580,625 | 491,225 |
| MW 524 | | 524 | 19.95 | 0 | 21,836 | 1,068,034 | 396,264 | 127,148 |
| MW 464 | | 464 | 19.98 | 0 | 0 | 3,509,107 | 1,662,937 | 1,090,554 |
| 1,2,3,6-tetra-O-galloyl-Beta-D-Glucose: 787 => 635, 617(BP), 465, 301 | | 788 | 20.08 | 0 | 2,030,433 | 6,287,225 | 5,015,553 | 1,821,748 |
| MW 550 | | 550 | 20.12 | 0 | 0 | 0 | 1,278,705 | 0 |
| MW 470 | |  | 20.14 | 0 | 11,801,513 | 37,214,766 | 40,937,409 | 19,803,391 |
| MW 480 | |  | 20.21 | 0 | 453,421 | 4,312,536 | 1,392,050 | 2,008,405 |
| MW 540 | | 540 | 20.24 | 0 | 467,109 | 1,482,194 | 825,380 | 489,994 |
| MW 390 | | 390 | 20.27 | 0 | 0 | 20,518 | 769,127 | 460,637 |
| MW 626, m/z 625 | | 626 | 20.27 | 0 | 749,585 | 4,781,774 | 1,543,531 | 1,103,217 |
| MW 426 | | 426 | 20.27 | 0 | 2,149,650 | 8,560,730 | 7,618,362 | 3,305,721 |
| MW 390, m/z 389 | |  | 20.30 | 0 | 322,732 | 0 | 946,111 | 539,304 |
| MW 506 | | 506 | 20.32 |  | 8,807,273 | 2,970,434 | 20,164,307 | 7,540,394 |
| MW 388, 448, m/z 447 | | 388/448 | 20.39 | 0 | 5,917,196 | 1,059,774 | 1,190,698 | 987,004 |
| MW 542 | | 542 | 20.41 | 0 | 1,043,447 | 727,914 | 697,337 | 386,566 |
| MW 548 | | 548 | 20.44 | 0 | 0 | 0 | 5,042,620 | 0 |
| MW 622 | | 622 | 20.46 | 0 | 1,750,175 | 381,135 | 20,186,216 | 3,287,570 |
| MW 434, m/z 433 | | 434 | 20.50 | 0 | 67,183 | 761,775 | 581,340 | 459,128 |
| MW 506 | | 506 | 20.98 |  | 3,890,906 | 2,139,958 | 11,743,145 | 4,230,980 |
| MW 616 | | 21.1 | 20.99 | 0 | 666,957 | 6,536,283 | 2,326,815 | 1,940,491 |
| MW 478 | |  | 21.03 | 0 | 4,398,699 | 8,735,362 | 5,671,544 | 6,070,115 |
| MW 540 | | 540 | 21.03 | 0 | 541,031 | 524,543 | 324,905 | 145,072 |
| m/z 463, 300.3; glycoside, | | 616 | 21.04 | 0 | 1,534,293 | 4,502,495 | 1,627,494 | 2,108,177 |
| MW 610? (used only m/z 609) | | 610 | 21.28 | 0 | 0 | 2,446,420 | 2,636,263 | 633,607 |
| MW 550 | | 550 | 21.37 | 0 | 938,957 | 479,272 | 3,189,779 | 474,643 |
| Mw 574 | | 574 | 21.45 | 0 | 419,937 | 1,691,551 | 1,439,873 | 486,641 |
| MW 434, m/z 433 | | 434 | 21.62 | 0 | 231,116 | 1,995,219 | 1,148,340 | 1,109,873 |
| MW 388, 448, m/z 447 | | 388/448 | 21.63 | 0 | 451,903 | 1,659,099 | 1,321,441 | 678,263 |
| MW 598 | | 598 | 21.67 | 0 | 1,532,957 | 2,756,931 | 1,799,260 | 1,378,802 |
| MW 478 | |  | 21.72 | 0 | 1,254,990 | 4,204,902 | 2,453,864 | 2,902,725 |
| MW 640, m/z 639.3 | | 640 | 21.78 | 0 | 235,919 | 263,195 | 1,258,522 | 314,764 |
| MW 624 | | 624 | 21.79 | 0 | 125,096 | 0 | 3,490,652 | 464,776 |
| MW 304 | | 304 | 21.79 | 0 | 1,472,340 | 14,247,902 | 4,140,210 | 4,948,535 |
| MW 548 | | 548 | 21.79 | 0 | 0 | 0 | 4,372,015 | 0 |
| MW 522, m/z 521 | | 522 | 21.82 | 0 | 1,911,388 | 1,393,760 | 745,626 | 564,097 |
| MW 550 | | 550 | 21.85 | 0 | 11,923,611 | 7,821,335 | 9,273,381 | 3,805,731 |
| MW 542 | | 542 | 21.98 | 0 | 0 | 279,641 | 1,109,826 | 29,365 |
| MW 510 | | 510 | 22.13 | 0 | 958,743 | 1,479,626 | 955,801 | 657,138 |
| MW 464 (m/z 463 interference from MW 610) | | 464 | 22.13 | 0 | 5,028,020 | 6,787,972 | 4,690,913 | 2,798,912 |
| MW 524 | | 524 | 22.13 | 0 | 5,074,694 | 6,595,420 | 4,791,146 | 2,887,474 |
| MW 598 | | 598 | 22.14 | 0 | 2,416,997 | 6,756,212 | 4,725,427 | 3,472,082 |
| Mw 574; this is MW 514: m/z 573 => m/z 513(10%), 351 (100%); | | 574 | 22.15 | 0 | 24,663,853 | 1,667,528 | 23,317,836 | 3,397,009 |
| MW 514: m/z 351 is fragment of m/z 573 which was likely [M+59]- of MW 514: 573 => 513, 351 | | 514 | 22.16 | 0 | 3,003,434 | 129,018 | 5,026,284 | 1,317,610 |
| MW 550 | | 550 | 22.23 | 0 | 956,643 | 330,077 | 982,020 | 296,304 |
| MW 434, m/z 433 | | 434 | 22.25 | 0 | 514,696 | 1,159,275 | 1,174,813 | 733,657 |
| MW 446: Only in EH, 0502-11 | | 446 | 22.31 | 0 | 0 | 0 | 1,589,934 | 0 |
| MW 522, m/z 521 | | 522 | 22.40 | 0 | 1,366,380 | 891,266 | 700,973 | 506,371 |
| MW 436 | | 436 | 22.40 | 0 | 278,413 | 1,389,304 | 1,333,064 | 727,132 |
| MW 342 or MW 582 m/z 311 and 326 correlate with m/z 341 and 581, 521 | | | 22.42 | 0 | 5,960,570 | 6,549,064 | 3,894,005 | 5,260,215 |
| m/z 326; may be fragment of m/z 341 (MW 342) | 327 | | 22.42 | 0 | 1,482,733 | 1,999,239 | 1,118,855 | 1,502,051 |
| MW 582 or m/z 521 +  60 | | | 22.45 | 0 | 3,429,575 | 2,070,817 | 1,515,375 | 727,737 |
| m/z 311 (no m/ z333);  fragment ion of m/z  341, 326? | | | 22.47 | 0 | 674,061 | 1,044,278 | 119,353 | 711,862 |
| MW 640, m/z 639.3 | 640 | | 22.53 | 0 | 264,049 | 291,438 | 1,374,053 |  |
| MW 610 diglycoside of MW 302 aglycone | 610 | | 22.54 | 168,002 | 6,231,772 | 40,508,995 | 18,282,542 | 9,482,272 |
| MW 610 (used only m/z 609) | 610 | | 22.58 | 0 | 1,788,971 | 12,755,116 | 5,791,142 | 2,983,376 |
| MW 464 (m/z 463 interference from MW 610) | 464 | | 22.70 | 0 | 903,366 | 2,187,842 | 968,173 | 712,296 |
| MW 524 | 524 | | 22.70 | 0 | 924,438 | 1,925,260 | 682,713 | 485,224 |
| m/z 351; fragment of m/z 573 | 514 | | 22.79 | 0 | 0 | 0 | 894,539 | 110,754 |
| MW 550 | 550 | | 23.02 | 0 | 523,156 | 1,615,102 | 675,875 | 306,657 |
| MW 548 | 548 | | 23.07 | 0 | 0 | 0 | 1,019,188 | 0 |
| MW 542 | 542 | | 23.08 | 0 | 345,860 | 360,276 | 3,377,988 | 1,909,624 |
| MW 388, 448, m/z 447 | 388/448 | | 23.09 | 0 | 469,076 | 2,042,327 | 1,358,277 | 544,744 |
| MW 478 |  | | 23.14 | 88,968 | 4,745,486 | 76,749,279 | 47,188,831 | 36,017,071 |
| Ellagic Acid | 302 | | 23.15 | 0 | 94,630,881 | 244,618,655 | 213,216,060 | 139,808,491 |
| MW 524 | 524 | | 23.18 | 56,711 | 664,559 | 211,286 | 385,609 | 128,993 |
| MW 778 Triglycoside: | 778 | | 23.19 | 0 | 6,523,263 | 783,377 | 3,899,504 | 1,817,891 |
| MW 610 (used only m/z 609) | 610 | | 23.25 | 0 | 71,582 | 0 | 1,384,398 | 16,400,956 |
| MW 426 | 426 | | 23.28 | 0 | 2,064,917 | 1,857,963 | 1,059,980 | 1,048,429 |
| MW 464 | 464 | | 23.42 | 0 | 724,293 | 318,573 | 250,096 | 115,827 |
| MW 404 | 404 | | 23.43 | 0 | 2,338,370 | 2,473,031 | 1,541,392 | 1,180,705 |
| MW 610 (used only m/z 609) | 610 | | 23.69 | 0 | 0 | 0 | 2,971,759 | 0 |
| Corniside II: 541 => 169 | 542 | | 24.06 | 0 | 332,694,673 | 413,076,711 | 308,383,444 | 248,276,736 |
| MW 658 | 658 | | 24.07 | 0 | 6,701,016 | 3,193,682 | 23,497,828 | 7,649,139 |
| MW 388, 448, m/z 447 | 388/448 | | 24.23 | 0 | 460,182 | 2,321,219 | 1,390,028 | 1,358,869 |
| MW 624 | 624 | | 24.37 | 0 | 1,318,920 | 0 | 0 | 78,956 |
| MW 658 | 658 | | 24.74 | 0 | 7,979,353 | 8,448,398 | 33,034,613 | 14,155,652 |
| MW 388, 448, m/z 447 | 388/448 | | 24.95 | 0 | 24,097 | 0 | 1,536,798 | 896,973 |
| MW 304 | 304 | | 25.17 | 0 | 541,688 | 894,837 | 483,510 | 491,081 |
| MW 550 | 550 | | 25.31 | 0 | 0 | 0 | 1,438,853 | 0 |
| MW 778 Triglycoside: | 778 | | 25.41 | 0 | 5,521,472 | 1,585,064 | 8,843,256 | 4,586,741 |
| MW 388, 448, m/z 447 | 388/448 | | 25.68 | 0 | 0 | 0 | 1,954,688 | 0 |
| MW 542 | 542 | | 25.75 | 0 | 2,443,716 | 1,930,764 | 3,644,897 | 1,731,127 |
| MW 658 | 658 | | 25.78 | 0 | 974,234 | 764,019 | 5,604,556 | 1,738,055 |
| MW 550 | 550 | | 25.90 | 0 | 0 | 0 | 2,306,535 | 0 |
| MW 778 Triglycoside: | 778 | | 26.02 | 0 | 1,770,456 | 558,112 | 2,639,528 | 1,520,444 |
| MW 426 | 426 | | 26.04 | 0 | 15,832,640 | 42,031,291 | 24,256,434 | 9,902,637 |
| MW 524 | 524 | | 26.27 | 0 | 148,568 | 997,158 | 208,729 | 155,341 |
| MW 524 | 524 | | 26.72 | 0 | 17,051 | 633,227 | 17,372 | 52,915 |
| MW 388, 448, m/z 447 | 388/448 | | 26.73 | 0 | 0 | 0 | 1,842,884 | 0 |
| MW 446: Only in source 3, 0502-11 | 446 | | 26.73 | 0 | 0 | 0 | 52,161,106 | 0 |
| MW 338 | 338 | | 27.63 | 0 | 0 | 2,225,998 | 188,763 | 371,054 |
| MW 422 | 422 | | 27.73 | 0 | 2,587,657 | 4,096,894 | 2,960,374 | 1,548,763 |
| MW 446: Only in source 3, 0502-11 | 446 | | 28.30 | 0 | 0 | 0 | 6,035,358 | 0 |
| MW 624 | 624 | | 28.42 | 0 | 0 | 0 | 947,971 | 0 |
| MW 510 | 510 | | 28.43 | 0 | 1,166,105 | 1,504,603 | 938,686 | 623,611 |
| MW 446: Only in source 3, 0502-11 | 446 | | 29.40 | 0 | 0 | 0 | 2,494,494 | 0 |
| MW 460? Only in source 3, 0502-11 | 460 | | 29.46 | 0 | 0 | 0 | 23,379,596 | 0 |
| MW 432 | 432 | | 29.52 | 0 | 0 | 0 | 6,639,525 | 0 |
| MW 330 | 330 | | 32.92 | 0 | 1,409,045 | 88,795 | 16,086 | 173,826 |
| MW 358 | 358 | | 33.03 | 0 | 0 | 0 | 526,595 | 0 |
| MW 542 | 542 | | 33.38 | 0 | 2,910,126 | 4,689,338 | 3,615,832 | 1,082,652 |
| MW 330 | 330 | | 34.26 | 0 | 1,422,916 | 10,401,914 | 7,894,019 | 3,992,077 |
| MW 332 | 332 | | 34.28 | 0 | 2,096,472 | 648,296 | 285,284 | 134,016 |
| MW 312, has m/z 333 ion | 312 | | 35.63 | 0 | 5,226,607 | 2,298,230 | 1,885,998 | 592,262 |
| MW 330 | 330 | | 35.71 | 0 | 144,446 | 1,170,222 | 889,791 | 492,934 |
| MW 822 | 822 | | 38.22 | 0 | 0 | 0 | 16,539,165 | 2,771,192 |
| m/z 351 fragment of m/z 821 | 822 | | 38.27 | 0 | 174,416 | 0 | 2,358,552 | 598,512 |
| MW 822 | 822 | | 39.09 | 0 | 0 | 0 | 3,882,793 | 626,249 |
| m/z 351 fragment of m/z 821 | 822 | | 39.20 | 0 | 70,690 | 0 | 744,545 | 141,505 |
| MW 314 | 314 | | 39.28 | 0 | 102,404 | 1,286,551 |  | 169,281 |
| Mw 484 | 484 | | 41.36 | 0 | 165,497 | 12,333,201 | 8,573,063 | 182,399 |
| MW 470 |  | | 43.10 | 0 | 0 | 346,235 | 2,894,480 | 2,881,729 |
| MW 470 |  | | 43.52 | 0 | 0 | 0 | 1,224,023 | 0 |
| MW 478 |  | | 44.30 | 0 | 2,139,765 | 833,976 | 888,751 | 88,938 |
| MW 372 | 372 | | 46.21 | 0 | 1,077,480 | 1,420,589 | 4,976,088 | 28,469 |
| MW 367 May be MP impurity but samples are slightly higher | 367 | | 47.42 | 145,513 | 680,727 | 759,114 | 662,073 | 134,803 |
