# Supplementary material for: Chemical profile dataset of Cornus officinalis from multiple sources using HPLC/MS
Source: Data Brief. 2019 Aug 13;25:104401. doi: 10.1016/j.dib.2019.104401 (PMC6722233; doi:10.1016/j.dib.2019.104401)
Supplement: Multimedia component 2 [file mmc2.docx]

|  |  | Blank | Source 1 | Source 2 | Source 3 | Source 4 |
| --- | --- | --- | --- | --- | --- | --- |
| **Compound** | Avg RT | RT-Blank | RT-1 | RT-2 | RT-3 | RT-4 |
| MW 296 (296 - 134 = 162 u; glycoside) | 1.62 | nd | 1.60 | 1.64 | 1.60 | 1.63 |
| Tartaric Acid | 1.91 | nd | 2.03 | 2.02 | 1.55 | 2.03 |
| Malic Acid | 1.98 | nd | 1.83 | 2.15 | 2.12 | 1.81 |
| MW 412-A | 2.08 | nd | 2.03 | 2.07 | 2.20 | 2.03 |
| MW 192 | 2.09 | nd | 1.98 | 2.07 | 2.28 | 2.03 |
| MW 232 | 2.20 | nd | 2.11 | 2.07 | 2.41 | 2.21 |
| MW 250 | 2.22 | 2.08 | 2.27 | 2.07 | 2.28 | 2.25 |
| Malic Acid, methyl Ester | 2.52 | nd | 2.55 | 2.51 | 2.57 | 2.43 |
| MW 460 | 2.58 | nd | 2.59 | 2.51 | 2.64 | 2.57 |
| MW 272 (or fragment ion of MW 362) | 2.59 | nd | 2.47 | 2.63 | 2.61 | 2.66 |
| MW 304 | 2.60 | nd | 2.47 | 2.59 | 2.57 | 2.75 |
| Mw 362 | 2.66 | nd | 2.63 | 2.67 | 2.61 | 2.71 |
| 5-Hydroxymethyl-2-furaldehyde | 2.76 | nd | 2.71 | 2.83 | 2.57 | 2.94 |
| Gallic Acid | 2.85 | nd | 2.86 | 2.87 | 2.81 | 2.84 |
| MW 644 | 3.01 | nd | 3.19 | 3.17 | 2.53 | 3.14 |
| MW 344 | 3.06 | nd | 2.78 | 2.99 | 3.52 | 2.94 |
| MW 524 | 3.33 | nd | 2.90 | 3.13 | 3.61 | 3.66 |
| MW 162 | 3.59 | nd | 4.17 | 3.36 | 3.40 | 3.44 |
| MW 348 | 3.67 | nd | 3.37 | 4.09 | 3.56 | 3.66 |
| MW 478 | 3.84 | nd | 3.11 | 4.40 | 3.40 | 4.44 |
| MW 312: only m/z 311 | 3.93 | nd | 3.69 | 4.51 | 3.27 | 4.25 |
| Mw 484 | 4.04 | nd | 4.17 | 3.94 | 3.91 | 4.12 |
| MW 412-B | 4.13 | nd | 4.77 | 4.30 | 3.01 | 4.44 |
| MW 480 | 4.14 | nd | 4.07 | 4.51 | 3.74 | 4.25 |
| MW 514 | 4.18 | nd | 4.33 | 4.19 | 3.87 | 4.31 |
| Mw 172 | 4.26 | nd | 4.33 | 4.19 | 4.19 | 4.31 |
| MW 154 | 5.07 | nd | 5.20 | 4.95 | 4.99 | 5.15 |
| MW 138 | 7.94 | nd | 7.99 | 7.97 | 7.63 | 8.17 |
| MW 242 | 8.63 | nd | 8.65 | 8.61 | 8.86 | 8.41 |
| MW 514 | 8.88 | nd | 9.71 | 8.53 | 8.86 | 8.41 |
| MW 510; m/z 329 fragment ion) | 8.95 | nd | 9.06 | 8.83 | 8.91 | 9.00 |
| MW 296 | 9.45 | nd | 9.65 | 9.29 | 9.33 | 9.51 |
| MW 404, m/z 463 [M+59]- | 9.96 | nd | 10.13 | 10.08 | 10.04 | 9.57 |
| MW 138 | 10.09 | nd | 10.18 | 10.01 | 10.09 | 10.06 |
| MW 444 | 10.09 | nd | 10.18 | 9.90 | 9.89 | 10.40 |
| MW 404 or m/z 403 fragment ion | 10.59 | nd | 10.53 | 10.01 | 10.62 | 11.19 |
| MW 406; m/z 405 | 10.97 | nd | 10.91 | 11.11 | 11.10 | 10.76 |
| m/z 285 | 11.05 | nd | 11.07 | 10.97 | 11.06 | 11.10 |
| MW 388, 448, m/z 447 | 11.14 | nd | 11.12 | 11.21 | 11.06 | 11.15 |
| MW 474, m/z 473 | 11.17 | nd | 11.49 | 11.21 | 10.80 | 11.19 |
| MW 624 | 11.33 | nd | nd | 11.30 | 11.31 | 11.37 |
| MW 312 | 11.36 | nd | 11.54 | 11.25 | 11.35 | 11.28 |
| m/z 179 fragment of MW 312 | 11.36 | nd | 11.78 | 11.16 | 11.18 | 11.33 |
| m/z 179 fragment ion of MW 312 | 11.39 | nd | 11.78 | 11.16 | 11.18 | 11.42 |
| m/z 315 | 11.52 | nd | 10.96 | 11.02 | 12.08 | 12.03 |
| MW 568 (diglycoside form of morroniside likely) | 11.55 | nd | 11.12 | 11.95 | 12.01 | 11.10 |
| m/z 285 | 11.75 | nd | 11.90 | 11.72 | 11.77 | 11.60 |
| MW 388, 448, m/z 447 | 11.76 | nd | 11.66 | 11.76 | 11.77 | 11.86 |
| MW 450, m/z 449 | 11.77 | nd | 11.82 | 11.72 | 11.85 | 11.69 |
| MW 478 | 12.03 | nd | 12.17 | 11.64 | 12.08 | 12.24 |
| MW 444 | 12.20 | nd | 12.20 | 12.13 | 12.20 | 12.28 |
| MW 522, m/z 521 | 12.21 | nd | 12.54 | 11.87 | 12.12 | 12.32 |
| Morroniside: m/z 405 and 465 have different profiles | 12.30 | nd | 12.31 | 12.31 | 12.35 | 12.24 |
| m/z 507 fragment ion of MW 568 | 12.57 | nd | 12.65 | 12.53 | 12.58 | 12.52 |
| m/z 285 | 12.59 | nd | 12.61 | 12.57 | 12.58 | 12.60 |
| MW 388, 448, m/z 447 | 12.61 | nd | 12.65 | 12.42 | 12.70 | 12.68 |
| Loganic Acid | 12.88 | nd | 12.94 | 12.83 | 12.89 | 12.84 |
| MW 522, m/z 521 | 13.26 | nd | 13.32 | 13.27 | 13.19 | 13.24 |
| MW 514, has m/z 271 fragment ion | 13.32 | nd | 13.43 | 13.23 | 13.30 | 13.32 |
| MW 388, 448, m/z 447 | 13.33 | nd | 13.24 | 13.38 | 13.30 | 13.39 |
| m/z 285 | 13.36 | nd | 13.28 | 13.34 | 13.38 | 13.43 |
| Mw 484 | 13.51 | nd | 13.55 | 13.49 | 13.53 | 13.47 |
| MW 636, m/z 635.1 | 13.71 | nd | 13.84 | 13.75 | 13.49 | 13.75 |
| Mw 636 | 13.71 | nd | 13.84 | 13.75 | 13.49 | 13.75 |
| MW 314 | 13.72 | nd | 13.79 | 13.70 | 13.74 | 13.65 |
| MW 180 | 13.78 | nd | 13.84 | 13.70 | 13.79 | 13.80 |
| MW 180 (m/z 179, 215/217) | 13.80 | nd | 13.84 | 13.75 | 13.79 | 13.80 |
| Mw 492, m/z 491 | 14.01 | nd | nd | nd | 13.96 | 14.06 |
| m/z 315 | 14.28 | nd | 14.55 | 14.12 | 14.37 | 14.06 |
| MW 580 | 14.32 | nd | 14.35 | 14.37 | 14.23 | 14.33 |
| MW 684 | 14.33 | nd | 14.29 | 14.42 | 14.37 | 14.22 |
| MW 506 | 14.35 |  | 14.50 | 14.37 | 14.41 | 14.12 |
| MW 540 | 14.40 | nd | 14.40 | 14.37 | 14.41 | 14.43 |
| Mw 492, m/z 491 | 14.59 | nd | 14.50 | 14.56 | 14.64 | 14.64 |
| m/z 163 fragment ion of MW 296 | 14.69 | nd | 14.35 | 14.88 | 14.73 | 14.80 |
| MW 626, m/z 625 | 14.71 | nd | 14.91 | 14.61 | 14.69 | 14.64 |
| MW 522, m/z 521 | 14.75 | nd | 14.86 | 14.88 | 14.60 | 14.64 |
| Mw 296 (has a m/z 591 [2M-H]- | 14.83 | nd | 14.96 | 14.79 | 14.78 | 14.80 |
| MW 198 or m/z 197 fragemt ion | 14.91 | nd | 14.91 | 14.88 | 14.86 | 15.00 |
| Mw 492, m/z 491 | 15.05 | nd | 15.06 | 15.02 | 15.00 | 15.10 |
| MW 636, m/z 635.1 | 15.09 | nd | 15.06 | 15.06 | 15.04 | 15.20 |
| Mw 636 | 15.09 | nd | 15.06 | 15.06 | 15.04 | 15.20 |
| MW 478 | 15.12 | nd | 15.30 | 15.20 | 15.43 | 14.53 |
| 7-O-Methylloganic acid | 15.32 | nd | 15.30 | 15.29 | 15.34 | 15.36 |
| MW 390 | 15.32 | nd | 15.30 | 15.29 | 15.34 | 15.36 |
| Tellimagrandin I | 15.43 | nd | 15.99 | 15.11 | 15.60 | 15.00 |
| Mw 492, m/z 491 | 15.46 | nd | 15.49 | 15.42 | 15.47 | 15.45 |
| MW 308 (has m/z 343/345 ion) | 15.55 | nd | 15.54 | 15.51 | 15.60 | 15.56 |
| MW 450, m/z 449 | 15.68 | nd | 15.72 | 15.65 | 15.64 | 15.71 |
| MW 390 | 15.76 | nd | 15.72 | 15.79 | 15.73 | 15.81 |
| MW 390, m/z 389 | 15.81 | nd | 15.82 | 15.88 | 15.73 | 15.81 |
| MW 388, 448, m/z 447 | 15.96 | nd | 15.99 | 15.92 | 15.95 | 15.97 |
| MW 226 | 16.00 | nd | 16.08 | 15.92 | 15.99 | 16.02 |
| MW 326 | 16.01 | nd | 15.99 | 16.19 | 15.95 | 15.92 |
| MW 506 | 16.06 |  | 16.04 | 16.14 | 16.08 | 15.97 |
| MW 522, m/z 521 | 16.25 | nd | 15.95 | 15.92 | 16.58 | 16.55 |
| m/z 315 | 16.32 | nd | 16.60 | 16.23 | 15.86 | 16.60 |
| Sweroside | 16.40 | nd | 16.47 | 16.35 | 16.41 | 16.36 |
| MW 450, m/z 449 | 16.44 | nd | 16.60 | 16.23 | 16.58 | 16.36 |
| Mw 492, m/z 491 | 16.49 | nd | 16.60 | 16.51 | 16.45 | 16.41 |
| MW 198 or m/z 197 fragemt ion | 16.53 | nd | 16.26 | 16.60 | 16.66 | 16.60 |
| MW 636, m/z 635.1 | 16.53 | nd | 16.69 | 16.51 | 16.70 | 16.22 |
| Mw 636 | 16.53 | nd | 16.69 | 16.51 | 16.70 | 16.22 |
| MW 326 | 16.58 | nd | 16.69 | 16.51 | 16.58 | 16.55 |
| MW 314 | 16.62 | nd | 16.69 | 16.64 | 16.53 | 16.60 |
| MW 464 | 16.65 | nd | 16.13 | 15.92 | 15.78 | 18.75 |
| MW 666, m/z 665.2 | 16.68 | nd | 16.69 | 16.60 | 16.62 | 16.81 |
| MW 666 | 16.68 | nd | 16.69 | 16.60 | 16.62 | 16.81 |
| Mw 492, m/z 491 | 16.81 | nd | 16.93 | 16.74 | 16.75 | 16.81 |
| MW 482 or MW 704 | 16.83 | nd | 16.89 | 16.69 | 16.84 | 16.90 |
| MW 552 | 16.87 | nd | 16.84 | 16.88 | 16.84 | 16.90 |
| MW 358 isomer | 16.91 | nd | 16.89 | 16.88 | 16.92 | 16.95 |
| MW 426 | 17.07 | nd | 17.05 | 16.92 | 17.34 | 16.95 |
| MW 390 Loganin; see below | 17.16 | nd | 17.27 | 17.18 | 17.04 | 17.15 |
| Loganin, m/z 449 BP | 17.21 | nd | 17.27 | 17.10 | 17.22 | 17.23 |
| MW 560 (m/z 595 [M+Cl]-) | 17.40 | nd | 17.45 | 17.36 | 17.41 | 17.39 |
| Mw 492, m/z 491 | 17.41 | nd | 17.45 | 17.36 | 17.45 | 17.39 |
| MW 624 | 17.50 | nd | 17.53 | 17.57 | 17.41 | 17.48 |
| MW 506 | 17.55 |  | 17.62 | 17.49 | 17.53 | 17.57 |
| MW 522, m/z 521 | 17.57 | nd | 17.66 | 17.44 | 17.62 | 17.57 |
| MW 482 or MW 704 | 17.59 | nd | 17.53 | 17.53 | 17.66 | 17.62 |
| MW 474, m/z 473 | 17.63 | nd | 17.66 | 17.57 | 17.49 | 17.81 |
| MW 552 | 17.65 | nd | 17.62 | 17.62 | 17.66 | 17.71 |
| MW 668? m/z 667; hump | 17.66 | nd | 18.83 | 16.92 | 17.41 | 17.48 |
| MW 666, m/z 665.2 | 17.71 | nd | 17.75 | 18.19 | 17.41 | 17.48 |
| MW 666 | 17.71 | nd | 17.75 | 18.19 | 17.41 | 17.48 |
| MW 164 | 17.72 | nd | 17.71 | 17.66 | 17.74 | 17.76 |
| MW 326 | 17.74 | nd | 17.75 | 17.75 | 17.70 | 17.76 |
| MW 540 | 18.14 | nd | 18.20 | 18.19 | 18.09 | 18.08 |
| MW 552 | 18.19 | nd | 18.20 | 18.01 | 18.47 | 18.08 |
| MW 164 (m/z 199/201 ion) | 18.21 | nd | 18.29 | 18.15 | 18.17 | 18.22 |
| MW 478 | 18.25 | nd | 18.25 | 18.24 | 18.25 | 18.27 |
| MW 390, m/z 389 | 18.42 | nd | 18.47 | 18.37 | 18.44 | 18.40 |
| MW 390 | 18.42 | nd | 18.47 | 18.37 | 18.44 | 18.40 |
| MW 474, m/z 473 | 18.46 | nd | 18.25 | 18.60 | 18.55 | 18.44 |
| MW 138 or m/z 137 fragment ion | 18.49 | nd | 18.56 | 18.42 | 18.59 | 18.40 |
| MW 506 | 18.62 |  | 18.69 | 18.65 | 18.47 | 18.66 |
| MW 522, m/z 521 | 18.67 | nd | 18.78 | 18.69 | 18.63 | 18.57 |
| MW 308 (does not have m/z 343/345 ion) | 18.83 | nd | 18.92 | 18.83 | 18.78 | 18.79 |
| MW 390, m/z 389 | 18.87 | nd | 18.92 | 19.01 | 18.85 | 18.70 |
| MW 390 | 18.87 | nd | 18.92 | 19.01 | 18.85 | 18.70 |
| MW 164 | 18.88 | nd | 19.22 | 18.83 | 18.66 | 18.79 |
| MW 474, m/z 473 | 18.90 | nd | 18.96 | 18.92 | 18.85 | 18.88 |
| MW 560 (m/z 595 [M+Cl]-) | 18.91 | nd | 18.96 | 18.87 | 18.89 | 18.92 |
| MW 464 | 18.94 | nd | 19.55 | 18.83 | 18.63 | 18.75 |
| MW 540 | 18.96 | nd | 18.96 | 19.01 | 19.04 | 18.83 |
| MW 510 | 19.23 | nd | 19.28 | 19.21 | 19.26 | 19.17 |
| MW 480 | 19.25 | nd | 19.28 | 19.21 | 19.26 | 19.23 |
| MW 478 | 19.25 | nd | 19.28 | 19.21 | 19.21 | 19.29 |
| MW 522, m/z 521 | 19.36 | nd | 19.39 | 19.26 | 19.39 | 19.40 |
| MW 534 (2 isomers merged) | 19.37 | nd | 18.96 | 19.49 | 19.52 | 19.51 |
| MW 404 | 19.41 | nd | 19.44 | 19.44 | 19.35 | 19.40 |
| MW 372 | 19.41 | nd | 19.49 | 19.35 | 19.35 | 19.46 |
| MW 542 | 19.49 | nd | 19.55 | 19.44 | 19.52 | 19.46 |
| MW 474, m/z 473 | 19.51 | nd | 19.55 | 19.49 | 19.48 | 19.51 |
| MW 622 | 19.69 | nd | 19.96 | 19.39 | 19.93 | 19.46 |
| MW 478 | 19.70 | nd | 19.66 | 19.59 | 19.89 | 19.67 |
| MW 390 | 19.70 | nd | 19.71 | 19.64 | 19.65 | 19.81 |
| MW 390, m/z 389 | 19.73 | nd | nd | nd | 19.65 | 19.81 |
| MW 506 | 19.76 |  | 19.81 | 19.74 | 19.73 | 19.76 |
| MW 522, m/z 521 | 19.77 | nd | 19.86 | 19.84 | 19.48 | 19.91 |
| MW 524 | 19.95 | nd | 19.86 | 20.00 | 20.02 | 19.91 |
| MW 464 | 19.98 | nd | nd | 19.89 | 20.02 | 20.03 |
| 1,2,3,6-tetra-O-galloyl-Beta-D-Glucose: 787 => 635, 617(BP), 465, 301 | 20.08 | nd | 20.99 | 19.94 | 19.77 | 19.62 |
| MW 550 | 20.12 | nd | nd | nd | 20.12 | nd |
| MW 470 | 20.14 | nd | 20.58 | 19.84 | 20.12 | 20.03 |
| MW 480 | 20.21 | nd | 20.36 | 20.17 | 20.21 | 20.09 |
| MW 540 | 20.24 | nd | 20.31 | 20.17 | 20.26 | 20.21 |
| MW 390 | 20.27 | nd | 20.26 | 20.34 | 20.26 | 20.21 |
| MW 626, m/z 625 | 20.27 | nd | 20.31 | 20.17 | 20.21 | 20.39 |
| MW 426 | 20.27 | nd | 21.70 | 20.45 | 19.43 | 19.51 |
| MW 390, m/z 389 | 20.30 | nd | 20.42 | nd | 20.26 | 20.21 |
| MW 506 | 20.32 |  | 20.36 | 20.29 | 20.30 | 20.33 |
| MW 388, 448, m/z 447 | 20.39 | nd | 20.42 | 20.29 | 20.39 | 20.44 |
| MW 542 | 20.41 | nd | 20.42 | 20.39 | 20.39 | 20.44 |
| MW 548 | 20.44 | nd | nd | nd | 20.44 | nd |
| MW 622 | 20.46 | nd | 20.52 | 20.29 | 20.53 | 20.51 |
| MW 434, m/z 433 | 20.50 | nd | 20.58 | 20.39 | 20.53 | 20.51 |
| MW 506 | 20.98 |  | 21.05 | 20.92 | 20.96 | 20.97 |
| MW 616 | 20.99 | nd | 21.17 | 20.87 | 20.96 | 20.97 |
| MW 478 | 21.03 | nd | 21.11 | 20.98 | 21.01 | 21.03 |
| MW 540 | 21.03 | nd | 21.17 | 20.98 | 21.01 | 20.97 |
| m/z 463, 300.3; glycoside | 21.04 | nd | 21.11 | 20.92 | 21.16 | 20.97 |
| MW 610 (used only m/z 609) | 21.28 | nd | 21.23 | 21.22 | 21.26 | 21.42 |
| MW 550 | 21.37 | nd | 21.41 | 21.42 | 21.16 | 21.48 |
| Mw 574 | 21.45 | nd | 21.41 | 21.37 | 21.55 | 21.48 |
| MW 434, m/z 433 | 21.62 | nd | 21.53 | 21.74 | 21.45 | 21.74 |
| MW 388, 448, m/z 447 | 21.63 | nd | 21.64 | 21.54 | 21.79 | 21.55 |
| MW 598 | 21.67 | nd | 21.76 | 21.59 | 21.65 | 21.69 |
| MW 478 | 21.72 | nd | 21.76 | 21.59 | 21.84 | 21.69 |
| MW 640, m/z 639.3 | 21.78 | nd | 22.03 | 21.59 | 21.89 | 21.62 |
| MW 624 | 21.79 | nd | 21.47 | nd | 21.55 | 22.34 |
| MW 304 | 21.79 | nd | 21.82 | 21.74 | 21.79 | 21.80 |
| MW 548 | 21.79 | nd | nd | nd | 21.79 | nd |
| MW 522, m/z 521 | 21.82 | nd | 21.87 | 21.78 | 21.70 | 21.91 |
| MW 550 | 21.85 | nd | 21.87 | 21.83 | 21.84 | 21.85 |
| MW 542 | 21.98 | nd | 21.87 | 21.88 | 22.08 | 22.10 |
| MW 510 | 22.13 | nd | 22.19 | 22.04 | 22.17 | 22.10 |
| MW 464 (m/z 463 interference frm MW 610) | 22.13 | nd | 22.14 | 22.09 | 22.13 | 22.16 |
| MW 524 | 22.13 | nd | 22.14 | 22.09 | 22.13 | 22.16 |
| MW 598 | 22.14 | nd | 22.19 | 22.09 | 22.13 | 22.16 |
| Mw 574; this is MW 514: m/z 573 => m/z 513(10%), 351 (100%); | 22.15 | nd | 22.19 | 22.09 | 22.17 | 22.16 |
| MW 514: m/z 351 is fragment of m/z 573 which was likely [M+59]- of MW 514: 573 => 513, 351 | 22.16 | nd | 22.19 | 22.04 | 22.17 | 22.22 |
| MW 550 | 22.23 | nd | 22.24 | 22.20 | 22.31 | 22.16 |
| MW 434, m/z 433 | 22.25 | nd | 22.29 | 22.20 | 22.27 | 22.22 |
| MW 446: Only in source 3, 0502-11 | 22.31 | nd | nd | nd | 22.31 | nd |
| MW 522, m/z 521 | 22.40 | nd | 22.50 | 22.41 | 22.36 | 22.34 |
| MW 436 | 22.40 | nd | 22.55 | 22.36 | 22.36 | 22.34 |
| MW 342 or MW 582; m/z 311 and 326 correlate with m/z 341 and 581, 521 | 22.42 | nd | 22.50 | 22.36 | 22.41 | 22.40 |
| m/z 326; may be fragment of m/z 341 (MW 342) | 22.42 | nd | 22.45 | 22.36 | 22.41 | 22.47 |
| MW 582 or m/z 521 + 60; m/z 521 done elsewhere | 22.45 | nd | 22.45 | 22.41 | 22.45 | 22.47 |
| m/z 311 (no m/ z333); fragment ion of m/z 341, 326 | 22.47 | nd | 22.45 | 22.41 | 22.55 | 22.47 |
| MW 640, m/z 639.3 | 22.53 | nd | 22.50 | 22.66 | 22.50 | 22.47 |
| MW 610 diglycoside of MW 302 aglycone | 22.54 | 22.87 | 22.67 | 22.46 | 22.55 | 22.47 |
| MW 610 (used only m/z 609) | 22.58 | nd | 22.67 | 22.51 | 22.55 | 22.58 |
| MW 464 (m/z 463 interference from MW 610) | 22.70 | nd | 22.55 | 22.72 | 22.69 | 22.83 |
| MW 524 | 22.70 | nd | 22.55 | 22.72 | 22.69 | 22.83 |
| m/z 351; fragment of m/z 573 | 22.79 | nd | nd | nd | 22.93 | 22.65 |
| MW 550 | 23.02 | nd | 23.07 | 22.96 | 23.02 | 23.02 |
| MW 548 | 23.07 | nd | nd | nd | 23.07 | nd |
| MW 542 | 23.08 | nd | 23.07 | 23.10 | 23.02 | 23.13 |
| MW 388, 448, m/z 447 | 23.09 | nd | 23.87 | 23.85 | 22.60 | 22.03 |
| MW 478 | 23.14 | 23.15 | 23.24 | 23.05 | 23.11 | 23.17 |
| Ellagic Acid | 23.15 | nd | 23.52 | 22.96 | 22.88 | 23.22 |
| MW 524 | 23.18 | 23.51 | 23.29 | 23.35 | 23.11 | 22.96 |
| MW 778 Triglycoside: | 23.19 | nd | 23.18 | 23.20 | 23.20 | 23.17 |
| MW 610 (used only m/z 609) | 23.25 | nd | 23.29 | 23.30 | 23.20 | 23.22 |
| MW 426 | 23.28 | nd | 23.47 | 23.35 | 22.55 | 23.73 |
| MW 464 | 23.42 | nd | 23.29 | 23.35 | 23.68 | 23.36 |
| MW 404 | 23.43 | nd | 23.47 | 23.40 | 23.43 | 23.42 |
| MW 610 (used only m/z 609) | 23.69 | nd | 23.71 | 23.62 | 23.68 | 23.73 |
| Corniside II: 541 => 169 | 24.06 | nd | 24.09 | 23.98 | 24.07 | 24.08 |
| MW 658 | 24.07 | nd | 24.12 | 24.05 | 24.04 | 24.05 |
| MW 388, 448, m/z 447 | 24.23 | nd | 24.56 | 24.64 | 23.90 | 23.83 |
| MW 624 | 24.37 | nd | 24.26 | nd | nd | 24.47 |
| MW 658 | 24.74 | nd | 24.78 | 24.69 | 24.74 | 24.75 |
| MW 388, 448, m/z 447 | 24.95 | nd | 25.56 | nd | 24.60 | 24.69 |
| MW 304 | 25.17 | nd | 25.26 | 25.09 | 25.15 | 25.17 |
| MW 550 | 25.31 | nd | nd | nd | 25.31 | nd |
| MW 778 Triglycoside: | 25.41 | nd | 25.43 | 25.39 | 25.36 | 25.44 |
| MW 388, 448, m/z 447 | 25.68 | nd | nd | nd | 25.68 | nd |
| MW 542 | 25.75 | nd | 25.81 | 25.64 | 25.74 | 25.81 |
| MW 658 | 25.78 | nd | 25.75 | 25.82 | 25.74 | 25.81 |
| MW 550 | 25.90 | nd | nd | nd | 25.90 | nd |
| MW 778 Triglycoside: | 26.02 | nd | 26.00 | 26.06 | 26.01 | 26.02 |
| MW 426 | 26.04 | nd | 26.31 | 25.88 | 25.96 | 26.02 |
| MW 524 | 26.27 | nd | 26.44 | 26.18 | 26.24 | 26.22 |
| MW 524 | 26.72 | nd | 26.71 | 26.69 | 26.69 | 26.77 |
| MW 388, 448, m/z 447 | 26.73 | nd | nd | nd | 26.73 | nd |
| MW 446: Only in source 3, 0502-11 | 26.73 | nd | nd | nd | 26.73 | nd |
| MW 338 | 27.63 | nd | nd | 27.40 | 27.85 | 27.64 |
| MW 422 | 27.73 | nd | 27.79 | 27.67 | 27.73 | 27.71 |
| MW 446: Only in source 3 | 28.30 | nd | nd | nd | 28.30 | nd |
| MW 624 | 28.42 | nd | nd | nd | 28.42 | nd |
| MW 510 | 28.43 | nd | 28.47 | 28.38 | 28.42 | 28.43 |
| MW 446: Only in source 3 | 29.40 | nd | nd | nd | 29.40 | nd |
| MW 460: Only in source 3 | 29.46 | nd | nd | nd | 29.46 | nd |
| MW 432 | 29.52 | nd | nd | nd | 29.52 | nd |
| MW 330 | 32.92 | nd | 32.91 | 32.91 | 32.88 | 32.96 |
| MW 358 | 33.03 | nd | nd | nd | 33.03 | nd |
| MW 542 | 33.38 | nd | 33.38 | 33.35 | 33.40 | 33.39 |
| MW 330 | 34.26 | nd | 34.58 | 34.10 | 34.17 | 34.17 |
| MW 332 | 34.28 | nd | 34.28 | 34.18 | 34.39 | 34.26 |
| MW 312, has m/z 333 ion | 35.63 | nd | 35.66 | 35.58 | 35.66 | 35.63 |
| MW 330 | 35.71 | nd | 35.66 | 35.71 | 35.73 | 35.72 |
| MW 822 | 38.22 | nd | nd | nd | 38.22 | 38.21 |
| m/z 351 fragment of m/z 821 | 38.27 | nd | 38.45 | nd | 38.15 | 38.21 |
| MW 822 | 39.09 | nd | nd | nd | 39.12 | 39.06 |
| m/z 351 fragment of m/z 821 | 39.20 | nd | 39.42 | nd | 39.12 | 39.06 |
| MW 314 | 39.28 | nd | 39.26 | 39.27 | 39.34 | 39.24 |
| Mw 484 | 41.36 | nd | 42.21 | 41.94 | 41.07 | 40.20 |
| MW 470 | 43.10 | nd | nd | 43.18 | 43.05 | 43.08 |
| MW 470 | 43.52 | nd | nd | nd | 43.52 | nd |
| MW 478 | 44.30 | ne | 44.28 | 44.23 | 44.25 | 44.44 |
| MW 372 | 46.21 | nd | 46.29 | 46.15 | 46.24 | 46.15 |
| MW 367 May be MP impurity but samples are slightly higher | 47.42 | 47.44 | 47.42 | 47.34 | 47.45 | 47.47 |
